# Included and excluded: an intersectionality-based policy analysis of young migrants’ vulnerability to sexual violence in Sweden

**DOI:** 10.1186/s12939-025-02454-x

**Published:** 2025-03-27

**Authors:** Tanya Andersson Nystedt, Tobias Herder, Anette Agardh, Benedict Oppong Asamoah

**Affiliations:** Department of Clinical Sciences Malmö Social Medicine and Global Health, Lund University, Jan Waldenströms gata 35, Malmö, 214 28 Sweden

**Keywords:** Migrants, Youth, Sexual violence, Intersectionality-based policy analysis, Sweden

## Abstract

**Introduction:**

Young migrants are increasingly recognised as being particularly vulnerable to sexual violence. This vulnerability is largely structural, due to the different laws and policies impacting their access to rights and services. Post-structural approaches to policy argue that policies are not responses to existing problems, but rather that social issues are constructed through policy. They define both whether something is a problem and how that problem is to be addressed. The aim of this study was to understand how sexual violence is conceived of in the Swedish policy environment and how this interacts with the migration regime to affect young migrants’ vulnerability to sexual violence.

**Methods:**

A systematic search was conducted to identify Swedish national-level policies: (1) addressing sexual violence; and (2) relating to the migration regime. 14 documents were included in the analysis: 6 addressed sexual violence, and 8 were related to migration. An intersectionality-based policy analysis was conducted by applying three questions to the relevant policies: (1) How is sexual violence conceived of in the policies? (2) How are young migrants represented in the policies? (3) How does the conception of sexual violence interact with the migration regime to impact young migrants’ vulnerability?

**Findings:**

Power is central to how sexual violence is conceived of in Swedish policies, as is access to human right and services. Young persons and migrants are both identified as vulnerable groups in these policies but as separate categories. Structural sources of vulnerability are recognised but not addressed in policies addressing sexual violence. The migration regime works largely to restrict migrants’ access to rights and services, thereby contributing to young migrants’ vulnerability to sexual violence.

**Conclusion:**

Although migrants and youth are included separately in policies addressing sexual violence, the migration regime largely works to exclude different groups of young migrants from access to rights and services and thereby increases their vulnerability to sexual violence.

## Background

Sexual violence is defined by the World Health Organization (WHO) as “any sexual act, attempt to obtain a sexual act, unwanted sexual comments or advances, or acts to traffic, or otherwise directed against a person’s sexuality using coercion, by any person regardless of their relationship to the victim, in any setting, including but not limited to home and work” [1]. Consent is critical to understanding sexual violence as the behaviour is not considered violence in itself; it becomes violence when it is unwanted or when the person is deemed unable to provide consent. Thus, sexual violence covers many different types of behaviours with varying degrees of physical contact and force. The distribution of vulnerabilities to and experiences of sexual violence in societies is unequal, as some groups have been shown to be more at-risk than others. The increased vulnerability of certain groups is evident in studies of sexual violence among young migrants, which have found both male and female migrants are particularly vulnerable to sexual violence [2, 3, 4]. It has been argued that structural factors, including laws and polices leading to limited access to human rights and services, including social and health services, have an impact on this vulnerability [2, 5, 6, 7].

Sexual violence is not only a determinant of health, as it has important implications for mental, physical and sexual and reproductive health outcomes [8], but it is also a health outcome resulting from an interplay of vulnerabilities at all levels of the socioecological model including the structural or policy level [9]. Traditional social and health policy analysis commonly regards policies as neutral constructs developed to address a given social problem and intended to benefit everyone equally. However, post-structuralist approaches have argued that social issues can be constructed through policy in that they define both whether something is a problem or not, and how that problem is to be addressed [10, 11].

One post-structural approach, the intersectionality-based policy analysis (IBPA) framework, posits that policies also signal who is important (and who is not) by which perspectives are included (or excluded) and whether different groups experience benefits or burdens as a result of these policies [11, 12]. It can be used to understand how policy constructs the power and privilege of individuals and groups in relation to their social, economic and political status, health and well-being.

An intersectionality-based policy analysis (IBPA) can be used to understand how policies affect diverse populations; to identify who benefits and who is excluded from the goals, priorities and resource allocations [12]. It was originally developed to understand differential health effects of individual policies or policy processes and has been particularly useful for understanding health inequities among indigenous populations [13, 14], and other vulnerable populations including people with substance abuse disorders [15–17], people living with HIV [18, 19], people with life-limiting illness [20] and their carers [21]. It has also been used to investigate broader policy contexts such as in the Haintz et al.’s study [22] seeking to understand how diverse policies impact women’s reproductive decision making in Australia.

The IBPA takes a broad approach to public policy acknowledging it as outputs or statements of intent by governments to address public problems [11]. As such they can take many different forms including laws, strategies and action plans. The IBPA also takes a “healthy public policy” approach [23], acknowledging that health outcomes, including sexual violence, are influenced also by determinants from outside the realm of the health sector. This means that policies outside of the health sector have important health impacts [11] and can be included in the analysis. To understand health among migrants, the migration regime, defined by Villalonga-Olives and colleagues as “the system of laws, regulations, policies and institutions within each country” which has “a profound effect on the lives of migrants” [24] has been shown to be particularly important for sexual and reproductive health [25, 26] and sexual violence among young migrants [2, 5, 27].

The IBPA is based on 8 guiding principles which are central tenets of intersectionality [11].

- Intersecting categories: No single social category can be assumed to have primacy in understanding a human life or experience. Social categories include gender, sexual orientation, age, migrant status, religion, nationality, ethnicity, marital status, amongst others. Individuals are part of multiple categories, and these categories can change over time and place. This is in contrast to a gender perspective, for example, where sex and gender are given primacy as central determinants of health.
- Multilevel analysis: Policy effects must be understood within and between levels from the individual level to the broader structural levels.
- Power: Social categories are constructed by systems of power that have both structural and discursive elements. The discursive elements refer to whose knowledge and experiences are valued and included and whose are excluded. The focus is not only on domination and marginalisation but also on the processes by which they are reproduced.
- Reflexivity: Recognising the diversity of experience and the existence of multiple truths.
- Time and space: Privilege and power are not universal; they are relational and contextual. The same person can experience both power and oppression depending on the situation or time, meaning that they can be marginalised in one context yet have power in another.
- Diverse types of knowledge: Includes the perspectives and diverse types of knowledges of those often marginalised or excluded.
- Social justice: Involves interrogating and challenging unfair and unequal power relations at the source.
- Equity: The focus on equity is a focus on fairness and/or targeting the most vulnerable or those subject to unjust and unfair treatment.

These principles are not mutually exclusive and are intended to be used together with the twelve questions that comprise the IBPA, five descriptive and seven transformative [11, 12]. Simplicity and flexibility are key features of the framework. It allows the application of all or some of the questions as relevant for the analysis in question. This paper applies three of the questions (see below) and will not include the full list which can be found elsewhere [11, 12].

Previous critical policy research investigating migrant representation in Swedish policies has found that they “reproduce, maintain, and normalize racial otherness and social exclusion” in the feminist policy to reduce men’s violence against women [28] and in SRHR policies [25], neglecting the power structures that impact the lived experiences of migrants. This study focuses on the broader policy context to understand how different policies interact to affect young migrants’ vulnerability to sexual violence.

## Methods

### Aim

This study aims to understand how sexual violence is conceived of in the Swedish policy context and how this conception interacts with other relevant policies to affect young migrants’ vulnerability to sexual violence. The term “young migrants” in this study refers to migrants of any gender aged between approximately 12 to 30 years. This policy analysis could contribute to the identification of specific sub-groups of migrants that may be at greater risk of sexual violence as well as to the understanding of the structural sources of that vulnerability. Such knowledge could be used to target particularly vulnerable groups with appropriate interventions and services.

### Identification of policy documents

There is no single policy addressing sexual violence in Sweden nor is sexual violence included in any migration policy. In line with previous studies investigating sexual health and migration in Sweden [25], relevant policies were identified through a search of online databases, including the webpages of the Public Health Agency of Sweden, the Migration Authority, and the Government Offices of Sweden. Broad and inclusive search terms were used including “migrants”, “migration”, or “sexual violence” resulting in 514 documents. Initial screening resulted in the removal of duplicates and documents that clearly did not qualify as policies. Additional relevant documents were identified through the existing policies. This resulted in 56 documents. These documents were further screened for relevance to migrants and sexual violence after which 20 documents were excluded leaving 36 for a full-text review (see the Appendix for a full list of policies included in the full-text review). Only policies currently in effect were included. Documents not directly relevant to sexual violence or young migrants’ access to rights and services were excluded at each stage as were supporting documentation for included policies (e.g. reports feeding into policies and propositions that later become laws). Acts that resulted in changes to existing included laws were not included separately. The first author conducted both the policy identification and screening. The process was discussed with all co-authors at each stage.

**Fig. 1:**
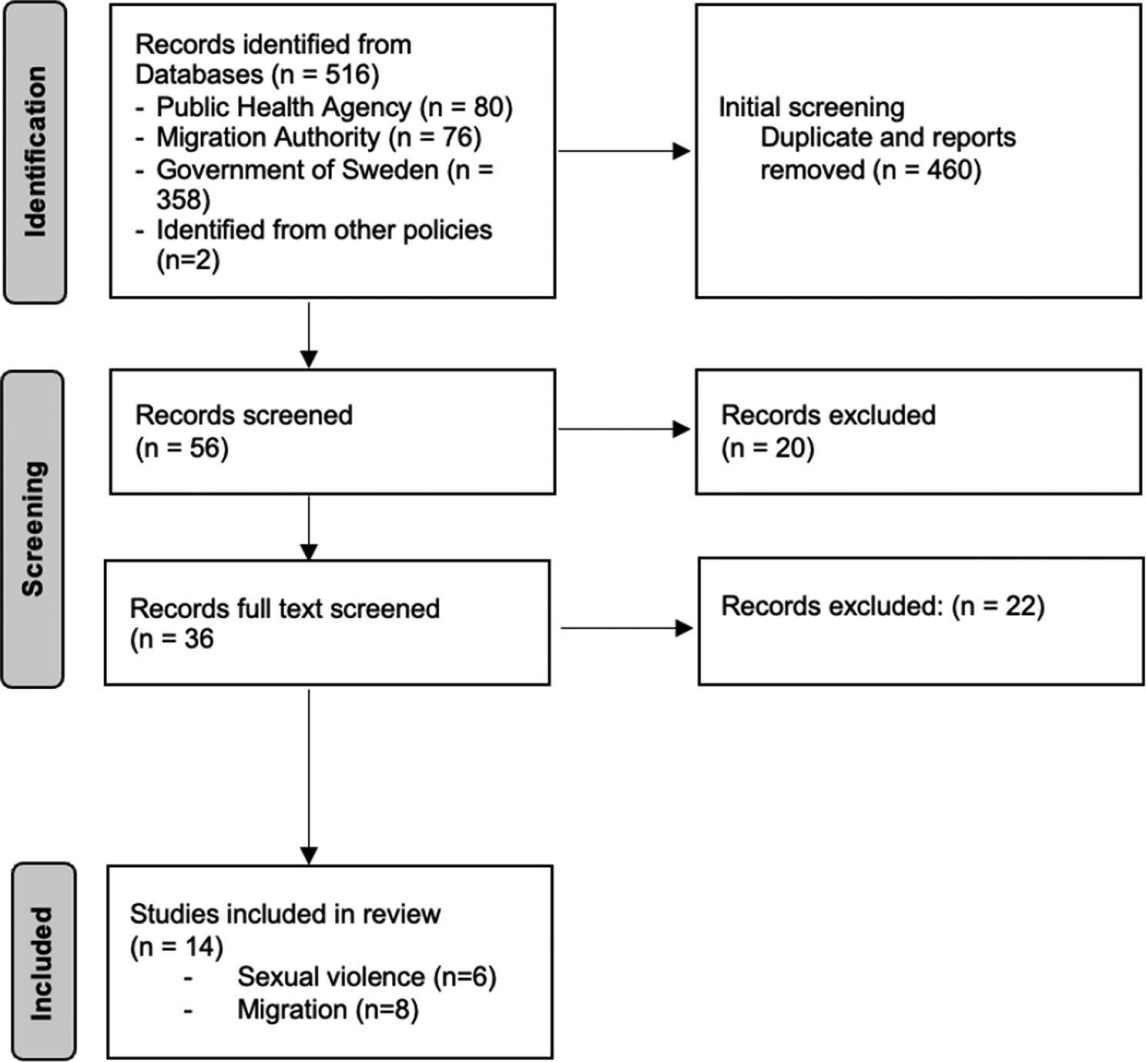
Policy selection process for inclusion in IBPA. Documents excluded if not directly relevant to sexual violence or young migrants’ access to rights and services

This resulted in 6 documents addressing sexual violence and 8 pertaining to the migration regime. See Fig. 1 for the policy selection process.

### Analysis

Of the 12 IBPA questions, 3 were selected as being relevant for the analysis of a policy context, as opposed to individual policies. These were:

1. What is the “problem” under consideration in this policy? Specifically, how is sexual violence conceived of in Swedish policies? (Question 2 in the IBPA Framework)
2. How are groups differentially affected by this representation of the problem? In other words, how are young migrants represented in the different policies? (Question 4 in the IBPA Framework)
3. What inequities actually exist in relation to “the problem”? Specifically, how does the conception of sexual violence interact with the migration regime to affect young migrants’ vulnerability? (Question 6 in the IBPA Framework)

All policies were analysed in their entirety. Sexual violence policies were analysed inductively using a coding frame based on questions 1 and 2 of the IBPA framework. Migration policies were analysed deductively applying questions 2 and 3 based on vulnerability factors identified from previous research, including access to rights and services. Analysis was facilitated by the use of NVivo release 14.23.0 and Excel version 16.90.2.

## Findings and discussion

The policy review identified 6 policy documents addressing sexual violence and 8 addressing young migrants’ access to rights and services (see Table 1). The full Swedish titles of the policies are presented in the Appendix.

**Table 1:** Summary of policy documents

Policy Document	Institution	Purpose
**Primary Policy Documents Addressing Sexual Violence**		
The National Strategy Against Men’s Violence Against Women and Honour-Related Violence and Oppression (2017–2026)	Swedish Gender Equality Agency	Developed as part of the Gender Equality Policy (SKR 2016/17:10). 4 main goals: prevention of violence against women, improved identification of violence and protection of victims, more effective law enforcement response, improved knowledge and methods.
The National Strategy for Sexual and Reproductive Health and Rights (SRHR) – Good, fair and equal sexual and reproductive health throughout the population (2020)	Public Health Agency of Sweden	Overarching goal: Good and equitable SRHR in the entire population. Sub-goals: (1) sexuality and sexual health - the freedom to decide if when and how they want to be sexually active and to choose their own sexual partners with consent; (2) Reproduction and reproductive health - choose if, when, how many and in what way they want to have children; (3) Empowerment, integrity and identity - strengthen empowerment and bodily integrity. Freedom from discrimination, harassment, violence (including sexual) and oppression; (4) Equal and equitable relationships - right to choose if, when and with whom they want to have a relationship or live with. Also the right to choose to enter into or terminate a marriage.
The National SRHR Action Plan (2023–2033)	Public Health Agency of Sweden	6 priority areas: (1) further develop SRHR as a part of public health; (2) strengthen SRHR promotion and prevention; (3) improve SRHR knowledge in the population; (4) promote equitable access to care, support and treatment services; (5) increase coordination within the SRHR sector; (6) strengthened knowledge generation and monitoring of SRHR.
Action Plan for Equal Rights and Opportunities for LGBTQI People (2020–2023)	Ministry of Employment	8 focus areas: (1) violence, discrimination and other violations; (2) young LGBTQI persons; (3) healthcare and social services; (4) private and family life; (5) culture; (6) civil society; (7) working life; and (8) elderly LGBTQI.
National Action Plan against Prostitution and Trafficking in Human Beings	Ministry of Health and Social Affairs	8 focus areas: (1) improved coordination; (2) strengthen prevention; (3) improve detection; (4) changes to the criminal code; (5) improved support and protection; (6) improve criminal justice response; (7) develop knowledge and capacity; (8) international coordination.
The Swedish Criminal Code	Ministry of Justice	Outlines criminalised sexual violence and possible consequences of the same.
**Migration Regime Documents**		
The Asylum Seeker Reception Act (1994:137)	Migration Authority	Describes the support that can be provided for asylum seekers or persons in need of protection who do not have the right to access social services. The support that can be provided is accommodation within the Migration Authority facilities or an accommodation allowance, daily subsistence allowance and an extra allowance (emergency money) for particular necessities.
The Social Services Act (2001:453)	Municipalities	Promotes financial and social security, equality in living conditions and social participation. Municipialities do this through providing financial support to those unable to sustain themselves as well as addressing particularly vulnerable groups including: children and young people, victims of crimes, the elderly, people with disabilities, people with substance abuse disorders, and people caring for relatives.
The Guardian for Unaccompanied Migrant Children Act (2005:429)	Migration Authority and Social Services	Specifies that unaccompanied migrant children under the age of 18 years should be provided with a legal guardians.
The Aliens Act (2005:716)	Migration Authority	Outlines the different ways and under which conditions foreign-born persons can have right to residence and work in Sweden.
The Aliens Ordinance (2006:97)	Migration Authority	Outlines more details on visa and residence permits as well as rules about the use of coercive measures including rejections and deportations.
The Healthcare for Asylum Seekers Act (2008:344)	Regions	Provides asylum seekers with the right to healthcare, including dental care, which cannot be deferred, maternity care, abortion care and contraceptive counselling.
The Healthcare for Foreigners Residing in Sweden without Necessary Permits Act (SFS 2013:407)	Regions	Provides undocumented migrants with the right to healthcare, including dental care, which cannot be deferred, maternity care, abortion care and contraceptive counselling.
The Upper Secondary School Act (2017:353)	Migration Authority	A temporary law in place until 20 December 2023 and is of particular relevance to young migrants as it allows for temporary residence permits for migrants who have not yet turned 25 years and are enrolled in full-time upper secondary education and valid up to 6 months after graduation.

### Question 1: how is sexual violence conceived in Swedish National policies?

All of the policies addressing sexual violence highlight the role of power and most raise both norms and access to human rights as factors of relevance for the vulnerability to sexual violence. Table 2 summarises the different approaches to sexual violence adopted by the different policies.

**Table 2:** Theoretical approaches adopted by the included policies addressing sexual violence and how they conceive of sexual

Policies	Approach	Definition	Sexual violence is due to:
The National Strategy Against Men’s Violence Against Women and Honour-Related Violence and Oppression (2017–2026)	Sex- and gender-based approach	Sex and gender categories and identities are given primacy to understand privilege and discrimination.	Power imbalances due to patriarchy and masculinity norms
The National Strategy for Sexual and Reproductive Health and Rights (SRHR) – Good, fair and equal sexual and reproductive health throughout the population (2020) and The National SRHR Action Plan (2023–2033)	Social determinants of health	“The conditions in which people are born, grow, work, live, and age, and the wider set of forces and systems shaping the conditions of daily life. These forces and systems include economic policies and systems, development agendas, social norms, social policies and political systems” (29). These determinants range from the structural, community, interpersonal and individual levels.	Interactions between structural, relational and individual factors determine access to power and resources
Action Plan for Equal Rights and Opportunities for LGBTQI People (2020–2023)	Norm critical perspective	Shifts the focus from individuals who are considered “different” to focus on what we consider normal and what privileges are conferred to those who are perceived as conforming to this norm. It is often used to interrogate the binary model of gender and heteronormativity.	Harmful norms including masculinity norms and heteronormativity
National Action Plan against Prostitution and Trafficking in Human Beings	Intersectional perspective	Developed by Crenshaw (30) as an analytical framework to understand how individuals’ and groups’ different social and political identities interact to form unique combinations of discrimination and privilege.	Intersecting power structures associated with gender, sexuality, ethnicity and socio-economic status
The Swedish Criminal Code – Chap. 6	Criminalised sexual offences	Key concepts are age, power and consent.	

As the name indicates, the National Strategy Against Men’s Violence Against Women and Honour-Related Violence and Oppression (henceforth VAW Strategy) conceives of sexual violence primarily from a sex and gender-based perspective, by giving the gender category primacy in the understanding of sexual violence. Although the strategy acknowledges that men, boys and LGBTQI persons can also be victims, this is clearly not the focus, and sexual violence is understood to be a result of power imbalances related to masculinity norms. The strategy does not distinguish sexual violence from other types of violence such as physical or psychological violence. The aim of this strategy is the right to bodily integrity and freedom from violence for all.

The VAW strategy is primarily focused on individuals, individual perpetrators and individual victims, to whom information can be provided as a way of preventing, identifying and responding to violence. The strategy acknowledges the role of unequal power relations between men and women as a contributing factor to violence while gender roles, stereotypes and masculinity norms are described as creating conditions that lead to violence. These are, however, not addressed in the interventions or actions suggested by the VAW strategy; instead, the more structural interventions are addressed as part of the broader Gender Equality Policy (not included in this study as it does not address sexual violence). The overarching goal of the Gender Equality Policy is that men and women should have the same power to influence society and their own lives. It addresses the structural determinants of vulnerability to sexual violence, but vulnerabilities resulting from other power differentials, such as age or migrant status, remain unaddressed.

The National Strategy for Sexual and Reproductive Health and Rights (henceforth SRHR Strategy) and the National SRHR Action Plan (henceforth SRHR Action Plan) both take a social determinants of health approach. The social determinants of health are the source of the unfair and avoidable differences in health [29]. This is a multi-level approach acknowledging that sexual and reproductive health and by extension sexual violence are a result of interactions between structural, relational and individual factors. The factors at different levels interact to influence to what extent an individual can access their rights. Access to rights can be mediated through factors such as socioeconomic status, ethnicity, disability status, age, sexual orientation and gender identity and migrant status, amongst others [29]. In these policies, experience of sexual violence is both a health outcome and a risk factor for additional negative SRHR outcomes such as contracting sexually transmitted infections (STIs) or unwanted pregnancies.

The Action Plan for Equal Rights and Opportunities for LGBTQI People (henceforth the LGBTQI Action Plan) takes a norm critical perspective developed in Sweden in the early 2000s and challenges the un-reflecting acceptance of what is considered “normal”, calling for an interrogation of the social norms in play and resisting categorisations into “us” and “them”. It conceives of sexual violence as the result of harmful norms such as heteronormativity and masculinity norms. An intersectional perspective is considered crucial to the application of this plan as LGBTQI are a heterogeneous group comprising different identities with different relations to social norms. One sub-section of this plan of particular relevance to this study relates to the asylum and residence permit seeking process for LGBTQI migrants in Sweden calling for migration officers to be competent on relevant rules, regulations, guidelines and case laws. A need for improved capacity in evaluation of the asylum applications, safety and security in refugee and migrant accommodation, as well as in reception and treatment of all types of residence applications is called for.

The National Action Plan against Prostitution and Trafficking in Human Beings (henceforth the Prostitution and Human Trafficking Action Plan) addresses sexual violence in that prostitution, the predominant form of human trafficking in Sweden, is considered a form of sexual violence. The purchase of sex is illegal in Sweden, while the selling of sex is not. Intersectional power structures associated with gender, sexuality, ethnicity and socio-economic status are considered to be key to understanding both prostitution and human trafficking. The focus of this plan is largely on decreasing demand through crime prevention programmes but also through addressing norms targeting primarily young men and boys including SRHR education and dissemination of gender equality information and messaging. Improved support and protection for victims are also described as critical to facilitate participation in criminal justice procedures and re-integration into society. This includes support for migrants lacking the right to stay in Sweden, enabling them to remain while deciding whether or not to participate in a criminal case as well as a programme to return trafficked persons to their home countries, run by the International Organisation for Migration (IOM). Support from civil society, social services and women’s shelters is particularly highlighted.

Finally, the criminal code focuses exclusively on criminalised sexual violence and the repercussions of the same. Here the key factor is consent which cannot be provided in the context of violence or threat of violence, if in a state unable to provide consent (when unconscious, under the influence of alcohol or drugs, etc.), or when the victim is dependent on the perpetrator. Another critical factor in the criminal code with regard to sexual violence is age, with any sexual activity with a person under the age of 15 years (the age of consent in Sweden) being considered rape or sexual exploitation of a child. An exception to this is selling sex where the age of consent is 18 years. Although the criminal code does not concern itself with understanding causes and vulnerabilities, it is clear that power is a central concept to the understanding of sexual violence given the importance of both age and the conditions under which consent can be given.

In fact, power differentials are central to the understanding of sexual violence in all of these policies. Norms and access to human rights are also critical concepts present in all the policies, with the exception of the criminal code. The lack of data on sexual violence in Sweden is also highlighted by all the policies except for the criminal code.

### Question 2: how are young migrants represented in the policies?

#### Sexual violence policies

The VAW strategy clearly identifies women and girls as the most disadvantaged group and the primary focus of the strategy. The strategy identifies specific sub-groups of women as particularly vulnerable including foreign-born women, young women and girls and persons with disabilities. Although these particularly vulnerable sub-groups are identified in the policy, there is a lack of an intersectional perspective and a deeper analysis of the different sources of vulnerabilities. For example, the VAW Strategy primarily addresses female migrants as a vulnerable group, overlooking variations within the migrant category (e.g. by age, legal status, etc.) and neglecting the vulnerability of young male migrants.

In addition, although the VAW Strategy, with its gender equality perspective, recognises that LGBTQI persons, men and boys can also be victims of sexual violence, it provides very little focus on them. Their invisibility can be seen as a barrier towards interventions targeting these groups, including identifying them as victims of violence and providing services. This is particularly problematic when addressing sexual violence experienced by young migrants, as studies suggest that such violence is more gender neutral and that young men and boys are also victims [2, 5, 31, 32]. It also compounds the already existing barriers for disclosure experienced by these groups [33].

The National SRHR Strategy and Action Plan explicitly take an intersectional perspective, recognising that vulnerable groups are heterogeneous and partially overlapping. They acknowledge that identities can change over time, that individuals may belong to one or more groups over their life course, and that groups that are considered vulnerable can change with changing social conditions and knowledge. Their focus is to strengthen the access to rights for vulnerable groups which include persons with migration experience and young persons but also people with low socio-economic status, LGBTQI persons, people with disabilities and elderly persons. The strategy and action plan propagate for universal interventions as well as interventions more targeted to vulnerable groups. In fact, in addition to the intersectional perspective, they also take an equity perspective and a gender equality perspective.

Migrants are a specific focus of the LGBTQI Action Plan, addressing the asylum process in particular, calling for it to be characterised by equal and fair treatment and adherence to the rule of law. The plan points out that homosexuality is still illegal in many countries and in some countries can even result in a death sentence. Even in countries where these laws are not enforced, they can result in violence and discrimination against LGBTQI persons. Therefore, fear of persecution due to sexual orientation or gender identity is a legitimate ground for seeking asylum. However, stigma and fear are also obstacles to using sexual orientation or gender identity as grounds for seeking asylum or to identifying their relevance in the asylum process.

Ethnic and racial minorities are also considered vulnerable, and the plan calls for synergy with the National Plans against racism. Honour-related norms are also perceived as being of importance to the LGBTQI community as they are often heteronormative and can result in families trying to “change” the young person’s sexuality through finding an “appropriate” partner or forcing a marriage.

The Prostitution and Human Trafficking Action Plan has a strong migration focus due to the focus on trafficking and particularly international trafficking. It highlights that a majority of persons selling sex in Sweden are migrant women. Another risk group identified are unaccompanied migrant children, most of whom are boys, at risk of selling sex. Children living in homes or residential care provided by social services are also considered at risk of selling sex, and many of these are young migrants.

The criminal code does not include any mention of vulnerable groups in the section on sexual violence, with the exception of children defined as under the age of 15 years for sexual activity and under the age of 18 years for selling sex.

All these policies, with the exception of the criminal code, recognise intersectional perspectives and the diversity of categories and vulnerabilities. However, an analysis of intra-categorical variability is largely absent. Also, although these policies adopt an understanding of structural vulnerabilities to sexual violence, these are not tackled in the policies addressing sexual violence.

#### The migration regime

None of the Swedish migration policies address sexual violence directly, but many restrict young migrants’ access to human rights and services considered critical in the policies addressing sexual violence. These restrictions in rights and services can serve to further the understanding of young migrants’ representation in the policy environment and in society.

### Access to rights

A key factor in determining access to rights and services and thereby young migrants’ vulnerability to sexual violence is their legal status [7]. Legal status in Sweden is regulated through a number of legal instruments including the Aliens Act (2005 − 716), the Aliens Ordinance (2006:97) and in the case of young migrants in particular, the Upper Secondary School Act (2017:353). A key factor here is that temporary residence permits have since 2016 (Act 2016:752) become the norm which does not allow access to social services and only very limited access to health services, both of which are considered critical to the response to sexual violence. Refugees, defined as persons who have had their grounds for seeking protection legally recognised, are an exception to this, as they are initially granted temporary residence but with full access to healthcare services and the possibility to apply for permanent residence after 3 years. Permanent residence permits are required in order to have full rights of access to social services and healthcare services.

The Asylum Seeker Reception Act (1994:137) is in place for persons who do not have the right to access social services and describes the support that can be provided for asylum seekers or persons in need of protection. The support that can be provided is accommodation within the Migration Authority facilities or an accommodation allowance, daily subsistence allowance and an extra allowance (emergency money) for particular necessities, such as winter clothing. This financial support can be reduced if the migrant refuses to participate in required activities or to cooperate in attempts to confirm their identities. The accommodation allowance can be withheld if the migrant chooses to reside in predefined socioeconomically challenged neighbourhoods (Act 2019:1204). These neighbourhoods generally have high proportions of inhabitants with migration backgrounds.

In the Aliens Act and Aliens Ordinance, age is a critical category, with a distinction made between being an adult (over 18 years) or being a child (under 18 years). Children under the age of 18 years have full access to both health and social services (including financial support) and the right to go to school, regardless of their legal status. Unaccompanied migrant children in particular have the right to secure accommodation in a “family-like environment” and the right to a legal guardian (Act 2005:439). Young migrants who have arrived as unaccompanied migrant children lose access to all the above-mentioned rights and services upon their 18th birthday unless they are granted permanent residence. As children and adults are treated differently with very different associated costs, age determinations of young migrants lacking identification documents have become a contentious issue [34]. Medical age determinations are prescribed in the Aliens Act despite their contested reliability and credibility with the risk of “imposing adulthood on minors” [35]. In fact, thousands of young persons in Sweden have been re-aged [35]. Unaccompanied migrant children who have been re-aged to adults also lose their right to the accommodations provided by the social services and need to be reassigned to Migration Authority accommodation which is often located far from schools and social support networks that the young migrant has established. If this re-aging happens in conjunction with a final rejection of the asylum application, the loss of rights is even more dramatic, as they are no longer eligible even for Migration Authority accommodation [35].

The Upper Secondary School Act (2017:353) is a temporary law of particular relevance to young migrants as it allowed (until 20 December 2023) for temporary residence permits for migrants who have not yet turned 25 years and are enrolled in full-time upper secondary education and valid up to 6 months after graduation. Permanent residence can be achieved if the young migrant can find employment sufficient to sustain them within this period. As part of this act, young migrants were not eligible for financial support under the Asylum Seeker Reception Act nor the Social Services Act. They are eligible for the very limited student grant (SEK1250) provided to all students in upper secondary school in Sweden, plus the supplementary allowance (maximum amount SEK 855) resulting in a monthly allowance maximum of SEK 2105 (approx. USD 200) which is meant to cover all their needs, including housing. In addition, this limited financial support is not provided during periods when school is not in session [36] meaning that that these young migrants lack any source of financial support for two months during the summer (July and August) unless they are able to find employment. These young migrants are in a particularly precarious position and lack public sector support to meet their basic needs including secure accommodation, a factor that has been identified as being critical to young migrants’ vulnerability to sexual violence [5, 37].

In terms of employment, asylum seekers and unaccompanied migrant children over the age of 16 years have the right to work if they have identification papers and their asylum application is being processed in Sweden. However, they are not eligible to work in jobs requiring certified skills leaving only the unskilled labour market available. Getting jobs in this sector is much harder due to language requirements as well as the high youth unemployment and high general unemployment rate in Sweden [38].

Migrants, including young migrants, who have gained residence on the basis of a romantic relationship often remain in violent relationships for fear of losing their temporary residence permits, despite provisions in the Aliens Act for persons exposed to intimate partner violence [39]. In addition, residence applications after the termination of a romantic relationship upon which the residence is based are more likely to be successful if the applicant is employed and able to support themselves and their dependents. This means that it is those who are already vulnerable and dependent on their perpetrators who are less likely to be granted residence. It also means that those who are most at risk of sexual violence are most likely to remain so under this migration regime.

The Aliens Ordinance (2006-97) also describes situations in which migrants can be detained, either during the asylum application process or in preparation for deportation. Conditions in reception and detention centres have been raised as contributing to young migrants’ risk of sexual violence [6, 40, 41, 42] and may be presumed particularly risky for adolescents over the age of 18 years who are housed with adult men of all ages. Detention and deportation are enacted by the police. Due to the insecure legal status of many young migrants, they are less likely to report criminalised violence to the police [43], including experiences of sexual violence [5, 43]. This means that many groups of young migrants lack legal protection from sexual violence and other forms of criminalised violence [43, 44].

### Access to healthcare and social services

The Social Services Act *(2001 − 453)* aims to promote financial and social security, equality in living conditions and social participation. In essence it is meant to address structural inequalities and vulnerabilities, including those identified as contributing to vulnerability to sexual violence. The social services provide financial support to those who are unable to maintain a reasonable standard of living as defined in that municipality. However, migrants lacking permanent residence permits are largely not eligible for support under the Social Services Act.

The Social Services Act is implemented at the municipal level, which means that it is the municipality of residence that is responsible for assessing eligibility for support and for its provision. This means that different municipalities may make different determinations of young migrants’ eligibility for different support services. Certain municipalities may provide financial support for persons with temporary residence permits while others might allow migrants lacking permanent residence permits to access emergency money for food, for example. However, in the case of undocumented migrants and in accordance with the Aliens Act, the social services are required to share information about individual migrants with the police, the Swedish Security Service, or the Migration Authority if requested. This means that undocumented migrants might not access social service support even when available for fear of detention and deportation [5, 7].

The Act on Healthcare Services for Asylum Seekers (Act 2008:344) and the Act on Healthcare Services for Foreigners Staying in Sweden without Necessary Permits (SFS 2013:407) provide asylum seekers and undocumented migrants with the right to healthcare which cannot be deferred, maternity care, abortion care and contraceptive counselling. This is the minimum package of services to be provided, and the different regions implementing this law and providing these services can opt to provide additional services. There is, however, no clear definition of what constitutes healthcare services that cannot be deferred, which leads providers to make differing determinations and often results in reported denials of healthcare services [5, 26].

### Question 3: what inequities actually exist in relation to sexual violence among young migrants?

With the exception of the Criminal Code, all of the included policies addressing sexual violence acknowledge the critical role of power in understanding vulnerability to sexual violence as well as the important roles of access to both rights and services for the prevention and treatment of sexual violence. They also include both young persons and migrants as vulnerable groups or as being at particular risk for not being able to access their rights. This is in contrast to the migration policies, which largely operate to define access to rights and services for different groups of migrants, thereby actively limiting this access for certain groups, such as undocumented migrants. These restrictions could limit certain young migrants’ power and agency which in turn could contribute to their vulnerability to sexual violence. In this way, young migrants are both included in policies addressing sexual violence while simultaneously being excluded from accessing certain protective rights and services in migration policies.

### The role of power in vulnerability to sexual violence

Power differentials are intrinsic to all the policies addressing sexual violence in Sweden. The focus is often at the individual level, the power of the perpetrator over the victim. This power can be physical, but it can also be psychological or emotional, including threats, blackmail and fear, or economic in the case of dependence on the perpetrator. These policies acknowledge that this individual power is underpinned by more structural forms of power, primarily patriarchy and associated norms such as masculinity norms and heteronormativity. They also give primacy to gender as a category for understanding sexual violence. For example, the VAW Strategy was formulated as part of the National Gender Equality Strategy which aims to ensure that women and men have the same access to power and resources to influence their lives. Its point of departure is a feminist analysis of society where the focus is on the structures and processes that are the root cause of inequality and a binary understanding of gender. The LGBTQI Action Plan also applies a gender equality perspective acknowledging the importance of understanding gender differences in relation to rights and opportunities. However, it recognises that a binary approach to gender not only risks perpetuating gender roles and norms, but also excludes persons who do not identify themselves in terms of binary gender categories.

Although this gender-focused conception of power is undoubtedly relevant for young migrants’ vulnerability to sexual violence, we would argue that they neglect the more fundamental restriction of power experienced by young migrants as a result of the different migration policies relevant to different groups of migrants. One of these restrictions is that in many cases young migrants cannot decide themselves where to live, whether that be in family homes, care or support homes, in Migration Authority accommodations or migrant detentions. For some, this can result in unsafe housing situations increasing their vulnerability to sexual violence [5, 27]. Moreover, it is not uncommon for young migrants to be moved between different Migration Authority accommodations, requiring them to adapt to new living spaces, new people, new schools and new cities [45, 46], where they then need to develop new social networks, determine who is safe and can be trusted, and who cannot. Young migrants who either are not eligible or who do not want to avail themselves of state provided accommodation are left at the mercy of the Swedish housing market which is characterised by multiple barriers to entry including long waiting lists and high costs. Young migrants can find themselves staying on people’s sofas or sharing black-market accommodations paying high rents or living in crowded conditions. Homelessness is a growing problem among young migrants [5, 47].

Another critical limitation to power is the ability to be self-sufficient which is limited for many groups of young migrants. Subsistence allowances in all cases are low and at best only able to meet the most basic needs [5]. Young migrants who are eligible to work face many barriers to access to the labour market due to the high general unemployment rate in Sweden and the high youth unemployment rate in particular [38] compounded by language barriers and discrimination [48, 49]. Undocumented young migrants over the age of 18 lack access to the legal labour market, leaving only illegal means of supporting themselves such as black-market employment, criminal gangs or sex work [5, 50] all of which increase their vulnerability to sexual violence.

### Young migrants as heterogeneous categories

**Fig. 2:**
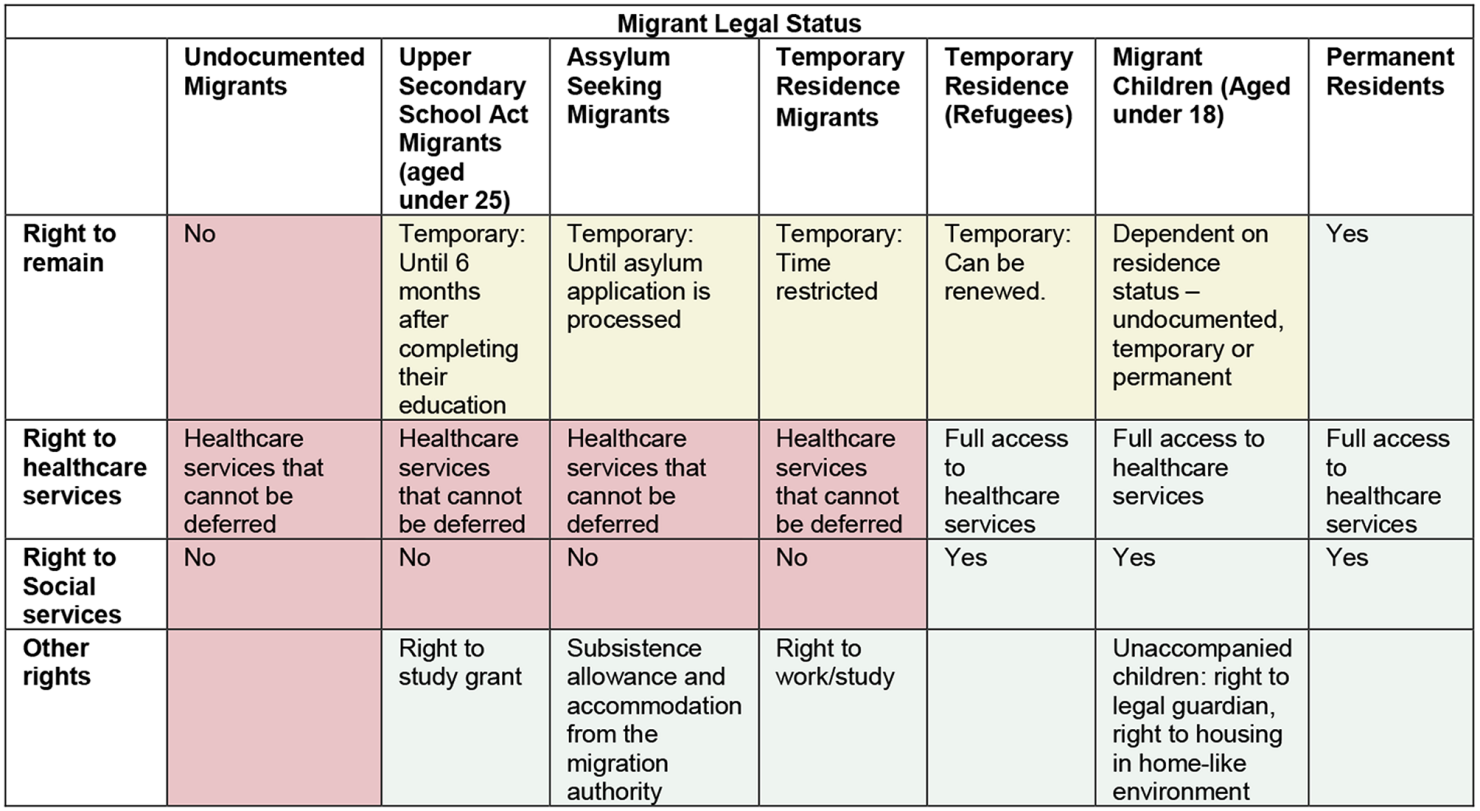
Access to the rights and services available to migrants in Sweden depending upon their legal status

Although the policies addressing sexual violence acknowledge that some groups of young migrants are more vulnerable to sexual violence than others (particularly female migrants and unaccompanied migrant children), migrants are treated largely as a single category. Intra-category variability and intersecting categories are largely not acknowledged. For example, youth are considered a vulnerable group as are migrants in all the strategies, but young migrants are largely not included under the assumption that their vulnerabilities would be captured either as young persons or as migrants. As with other intersecting categories, this assumption does not hold true. For example, access to different rights and services is the result of the combination of type of residence permits and age (under 18 or over 18 years) as illustrated in Fig. 2.

It is important to keep in mind that movement through each of these categories is not linear from lower access to rights and services to greater access to rights and services. For instance, an unaccompanied migrant child can become an adult asylum seeker when they turn 18, they may be conferred refugee status, they may have gain residence under the Upper Secondary School Act or even obtain permanent residence. They may also become undocumented migrants. Each of these changes is accompanied by an abrupt change in their access to rights and services, influencing their ability to meet their basic needs. In keeping with the tenets of intersectionality this clearly depicts how the vulnerability to sexual violence can change over time with changing legal statuses and the accompanying changes in access to rights and services. The impact of time on young migrants’ vulnerability also includes the duration of time spent as migrants with insecure legal statuses waiting for asylum processes, or as migrants under the Upper Secondary School Act, where they are required to be employed within 6 months of completing their educational programmes. Studies have suggested that these sometimes extended processes can lead to increased exclusion and social isolation of young migrants, accompanied by feelings of shame, loss of hope [35, 46] and vulnerability to sexual violence. Time is also critical to young migrants in particular because although the transition from childhood to adulthood in reality is a process, this happens legally from one day to the next upon one’s 18th birthday or from the moment they are re-aged to 18 years or older [51].

In the policies addressing sexual violence, gender as a category is given primacy, with women and girls seen as victims and men as perpetrators. This approach may be effective for understanding sexual violence in the population at large, where this pattern generally holds true [52]. However, it may be less appropriate for understanding vulnerability to sexual violence among young migrants, as young men and boys have been found to be vulnerable [31, 32], including in Sweden [3]. Some of the policies address this distinction through presuming that sexual violence is always the result of the same structures and norms as sexual violence in the general population (patriarchy, masculinity norms and heteronormativity), but this is not pursued further. Although these norms and structures undoubtedly play a role in young migrants’ vulnerability to sexual violence, the policies fail to consider the structural vulnerabilities they are exposed to, such as their legal status, and thus risk missing key pieces of the puzzle.

### Lack of knowledge

The knowledge gaps regarding sexual violence among young migrants are substantial as highlighted by various Swedish policies addressing sexual violence. Non-disclosure of sexual violence is high [53] and a substantial proportion of sexual violence remains unreported and unquantified [1]. Often violence is only identified if it is severe enough to require healthcare services, and even then, it is not always detected. Additionally, not all healthcare services are obligated to report identified sexual violence, such as primary healthcare services, meaning that not all cases requiring healthcare are reported and are subsequently not included in national statistics. The lack of evidence is substantial for many sub-groups, including young migrants, and available national data may not be disaggregated by country of birth or migration status. Regarding national official statistics based on reported cases, it is of relevance to consider the additional barriers to services and reporting for young migrants. These barriers include lack of trust in institutions, language barriers, and lack of access to particular services [5, 34, 43], which further hampers identification of victims of violence.

Information about race/ethnicity is almost entirely absent from existing data as Sweden does not collect that information in surveys or registers [54]. Instead, other indicators are used such as country of birth and parents’ country of birth to indicate migrant and/or foreign-born status which limits the ability to assess discrimination or vulnerability along ethnic lines. Race and ethnicity have been shown to be critical categories for understanding gender-based violence in other contexts [30] and could be key aspects for understanding young migrants’ vulnerability to sexual violence.

The lack of data on sexual violence is compounded in the case of young migrants, as there is a lower participation of migrants in public health surveys [55] but also because several groups of migrants are not represented in population-based surveys including asylum seekers and undocumented migrants. A recent study on professionals working with migrants also suggests that it is not uncommon that young migrants in Sweden are mobile, moving often and living in more ad hoc living situations [5]. This means that young migrants’ experiences are largely not captured in the limited data available on sexual violence in Sweden and thereby excluded from policy.

Table 3 shows a summary of the findings in relation to the three IBPA questions under consideration.

**Table 3:** Summary of findings

	IBPA Questions	Key Findings
1	How is sexual violence conceived in Swedish policies?	Power differentials are key to the understanding of sexual violence. Norms and access to human rights are also critical concepts. There is a lack of data on sexual violence
2	How are migrants represented in Swedish policies?	Migrants and youth are both identified separately as vulnerable populations in sexual violence policies but not migrant youth (except unaccompanied migrant children). Migration policies focus on access to specific rights and services determined by legal status
3	How does the conception of sexual violence interact with the migration regime to determine young migrants’ vulnerability to sexual violence	Power differentials can be a critical source of vulnerability for young migrants (including access to employment and housing) – beyond gender-based power differences. Young migrants access to rights and services and thereby vulnerability to sexual violence is predicated upon legal status and age. Knowledge about young migrants’ experiences and vulnerabilities to sexual violence is largely excluded from policies.

#### Methodological considerations

A key strength of the IBPA framework is the focus on intersectionality, on intersecting power structures that co-construct and constrain individuals’ possibilities in life, and the integration of power, equity and social justice in the analysis. Another strength of the IBPA framework is its flexibility, allowing for the application of all questions or a selection of questions. It can also be applied to understand a single policy or to understand a set of policies and their effects on health outcomes or determinants of health. A strength of this study is the novel application of the framework to gain an understanding of young migrants’ structural vulnerabilities to sexual violence in Sweden.

The current study does not purport to provide an exhaustive analysis of all migration policies in force in Sweden. However, it includes the key instruments and their implications for vulnerability to sexual violence and any potentially excluded document is likely to have only a limited effect, if any, on the findings.

One potential limitation of this study is the absence of a historical perspective. Instead, the migration and sexual violence policies are analysed as they are currently formulated, not how they have been developed and adapted over time. There have been substantial changes, especially to the migration regime, over the past several years, with increasingly restrictive policies curtailing migrants’ access to rights and services [56]. This was considered beyond the scope of this study and could be the focus of further research.

Lastly, this paper focuses only on national-level policies, but many critical services are the responsibility of the regional and municipal levels and their policies and instruments. This means, for example, that in certain municipalities, young migrants may be able to access more rights than in others, especially in cases where national-level policies contain contradictions (84). For example, the Social Services Act is implemented at the municipal level. It specifies that it is only relevant for persons with permanent residence permits; however, it also states that municipalities are responsible for the welfare of *all* their residents, some of whom may have temporary residence permits or be undocumented. This means that some municipalities provide certain social services to persons with temporary residence permits or even undocumented migrants while others do not. However, national level policies are still the guiding framework for work at the lower levels and there are indications that municipalities are taking an increasingly narrow approach to eligibility to services.

## Conclusion

This is the first study that we are aware of that investigates the effect of a policy context on constructing young migrants’ vulnerability to sexual violence. By applying an intersectional perspective, our policy analysis suggests that Swedish policies addressing sexual violence highlight the importance of power as well as the access to rights and services in constructing vulnerability to sexual violence. Migrants and youth are specifically included as particularly vulnerable groups with a particular focus on migrant women and unaccompanied migrant children (most of whom are boys). In contrast, different migration policies exclude certain young migrants from accessing certain rights and services, thereby diminishing their power and increasing their vulnerability to sexual violence. An intersectional perspective can be used to understand how young migrants’ vulnerability to sexual violence may change over time with transitions from childhood to adulthood, shifts in legal status and varying degrees of access to rights and services.

## Appendix – List of policy documents identified and reviewed

**Table:** 

	Document	Year
1	Brottsbalk (1962:700)	1962
2	Lag (1990:52) med särskilda bestämmelser om vård av unga	1990
3	Lag (1994:137) om mottagande av asylsökande m.fl.	1994
4	Förordning (1994:361) om mottagande av asylsökande m.fl.	1994
5	Förordning (1996:1357) om statlig ersättning om hälso- och sjukvård till asylsökande	1996
6	Lag (2001:82) om svenskt medborgarskap	2001
7	Socialtjänstlag (2001:453)	2001
8	Lag (2005:429) om god man för ensamkommande barn	2005
9	Utlänningslag (2005:716)	2005
10	Utlänningsförordning (2006:97)	2006
11	Lag (2008:344) om hälso- och sjukvård åt asylsökande m.fl.	2008
12	Förordning (2008:347) om hälso- och sjukvård åt asylsökande m.fl.	2008
13	Diskrimineringslag (2008:567)	2008
14	Förordning (2010:1122) om statlig ersättning för insatser för vissa utlänningar	2010
15	Svensk författningssamling – lag om hälso-och sjukvård till vissa utlänningar som vistas i Sverige utan nödvändiga tillstånd SFS 2013:407	2013
16	Våld i nära relationer – en folkhälsofråga: förslag för ett effektivare arbete (SOU 2014:49)	2014
17	Makt, mål och myndighet – feministisk politik för en jämställd framtid (Skr 2016/17:10)	2016
18	En nationell strategi för att förebygga och bekämpa mäns våld mot kvinnor (Skr 2016/17:10)	2016
19	Utvidgat hinder mot erkännande av utländska barnäktenskap (SOU 2017:96)	2017
20	Förordning (2017:193) om statlig ersättning för asylsökande m.fl.	2017
21	Lag (2017:353) om uppehållstillstånd för studerande på gymnasial nivå	2017
22	Promemoria – Långsiktigt reformprogram för minskad segregation år 2017–2025	2017
23	Nästa steg på vägen mot en mer jämlik hälsa: föreslag för ett långsiktigt arbete för en god och jämlik hälsa (SOU 2017:47)	2017
24	Regeringens proposition 2017/18:249: God och jämlik hälsa – en utvecklad folkhälsopolitik	2017
25	Handlingsplan mot prostitution och människohandel	2018
26	Lag (2018:1197) om Förenta Nationernas konvention om barnets rättigheter	2018
27	Handlingsplan för hbtqi-personers lika rättigheter och möjligheter	2020
28	På väg mot en god och jämlik hälsa: stödstruktur för det statliga folkhälsoarbetet	2020
29	Nationell strategi för sexuell och reproduktiv hälsa och rättigheter (SRHR): en god, jämlik och jämställd sexuell och reproduktiv hälsa i hela befolkningen	2020
30	Åtgärdsprogram mot afrofobi	2021
31	Åtgärdsprogram mot antisemitism	2021
32	Åtgärdsprogram mot antiziganism	2021
33	Åtgärdsprogram mot islamofobi	2021
34	Förordning (2022:257) om statsbidrag till kvinno- och tjejjourer och vissa andra ideella organisationer inom brottsofferområdet	2022
35	Förordning (2022:722) om statsbidrag för visst våldsförebyggande arbete	2022
36	Nationell handlingsplan för sexuell och reproduktiv hälsa och rättigheter (SRHR) i Sverige: genomförandet av den nationella-strategin 2023–2033.	2023

## Data Availability

Data is derived from Swedish policies as listed in the manuscript and publicly available.

## Abbreviations

IBPA: Intersectionality based Policy Analysis
IOM: International Organization for Migration
LGBTQI: Lesbian, Gay, Bisexual, Transgender, Queer, Intersex
SEK: Swedish Krona
SRHR: Sexual and Reproductive Health and Rights
STI: Sexually Transmitted Infections
VAW: Violence Against Women
WHO: World Health Organization
